# Development and validation of a prediction nomogram for a 6-month unfavorable prognosis in traumatic brain-injured patients undergoing primary decompressive craniectomy: An observational study

**DOI:** 10.3389/fneur.2022.944608

**Published:** 2022-08-03

**Authors:** Zhiji Tang, Kun Hu, Ruijin Yang, Mingang Zou, Ming Zhong, Qiangliang Huang, Wenjin Wei, Qiuhua Jiang

**Affiliations:** ^1^Department of Neurosurgery, Ganzhou People's Hospital, Ganzhou, China; ^2^Department of Neurosurgery, HuiChang County People's Hospital, HuiChang, China

**Keywords:** traumatic brain injury, decompressive craniectomy, nomogram, validation, prognosis

## Abstract

**Objective:**

This study was designed to develop and validate a risk-prediction nomogram to predict a 6-month unfavorable prognosis in patients with traumatic brain-injured (TBI) undergoing primary decompressive craniectomy (DC).

**Methods:**

The clinical data of 391 TBI patients with primary DC who were admitted from 2012 to 2020 were reviewed, from which 274 patients were enrolled in the training group, while 117 were enrolled in the internal validation group, randomly. The external data sets containing 80 patients were obtained from another hospital. Independent predictors of the 6-month unfavorable prognosis were analyzed using multivariate logistic regression. Furthermore, a nomogram prediction model was constructed using R software. After evaluation of the model, internal and external validations were performed to verify the efficiency of the model using the area under the receiver operating characteristic curves and the calibration plots.

**Results:**

In multivariate analysis, age(*p* = 0.001), Glasgow Score Scale (GCS) (*p* < 0.001), operative blood loss of >750 ml (*p* = 0.045), completely effaced basal cisterns (*p* < 0.001), intraoperative hypotension(*p* = 0.001), and activated partial thromboplastin time (APTT) of >36 (*p* = 0.012) were the early independent predictors for 6-month unfavorable prognosis in patients with TBI after primary DC. The AUC for the training, internal, and external validation cohorts was 0.93 (95%CI, 0.89–0.96, *p* < 0.0001), 0.89 (95%CI, 0.82–0.94, *p* < 0.0001), and 0.90 (95%CI, 0.84–0.97, *p* < 0.0001), respectively, which indicated that the prediction model had an excellent capability of discrimination. Calibration of the model was exhibited by the calibration plots, which showed an optimal concordance between the predicted 6-month unfavorable prognosis probability and actual probability in both training and validation cohorts.

**Conclusion:**

This prediction model for a 6-month unfavorable prognosis in patients with TBI undergoing primary DC can evaluate the prognosis accurately and enhance the early identification of high-risk patients.

## Introduction

The significant role of decompressive craniectomy (DC) in the treatment of severe traumatic brain injury (TBI) has been verified by a large number of clinical trials and experiences (1). To date, two famous multicenter randomized controlled trials (RCTs) have been conducted on the indication and effectiveness of DC in the treatment of TBI, both of which focus on patients undergoing secondary DC (2, 3). However, few studies have investigated primary DC as a point of interest. In emergency situations, such as a massive intracranial lesion, an observably progressive decline in the patient's consciousness, and even hernia, primary DC may be the only way to relieve high intracranial pressure (ICP) refractory to medical treatment and save the patient's life (4–6). However, a series of factors, such as critical condition at presentation, prompt change in status after admission, and insufficient preoperative preparation, have resulted in high postoperative mortality and unreasonable prognosis for some patients after primary DC; furthermore, most of the deaths occur in the early stage of the disease (7–11).

In our previous study on early predictors of 30-day mortality after primary DC in patients with TBI, we observed an early death rate of 30.8% (12). However, not all patients who survived the decompressive procedure showed a promising prognosis. Some patients fell into a vegetative state or even died of organ failure a few months later, which resulted in the placement of not only a heavy economic burden but also an extremely large psychological burden on the relatives of the patient (7, 8, 13). However, doctors can find it difficult to give a clear prognosis to patients after DC based only on their experience. Therefore, it is necessary to establish a model to predict patient prognosis early that can not only enable us to understand disease progression but also take reasonable interventional measures in the early stages after trauma to improve prognosis. In addition, the early prediction model should not only provide a reference for the decision-making of families with different cultures and beliefs but also encourage the rational allocation of medical resources.

As a continuation of our previous study, in the present study, we built an early (soon after DC) prediction model for the 6-month unfavorable clinical outcomes of patients with TBI after primary DC and then further validated the constructed model internally and externally so that it can be used conveniently in the clinic. The outcome variable we used was the Glasgow Outcome Scale (GOS) score (14). Notably, we only regarded death and vegetative state (GOS scores of 1 and 2, respectively) as unfavorable outcomes in this study. The reason for this classification is that severe disability seems to be more acceptable to the patient's family than a vegetative state (15).

## Methods

### Training and internal validation cohorts

Clinical data were obtained from the database of Ganzhou People's Hospital, Jiangxi Province, China. We retrospectively reviewed the records of 422 patients with primary DC from January 2012 to January 2020. For these patients, primary DC was performed as the first therapeutic procedure after the initial phase of resuscitation (usually <24 h). Our exclusion criteria were as follows: (1) since the object of the study is primary DC, secondary DC should not be considered; (2) loss to follow-up or incomplete data within 6 months; (3) abandonment by the guardians or accidental death; (4) posterior fossa decompression or craniocerebral penetrating injury; (5) severe underlying pathologies or brain stem injury; and (6) bilateral massive cerebral infarction and circulatory failure after admission. After applying these criteria, 391 patients were finally enrolled in this study.

The 391 selected patients were additionally divided into a training cohort (*n* = 274) and an internal validation cohort (*n* = 117) randomly at a ratio of 7:3. The training data were used to construct the nomogram, whereas the internal validation data were used to verify the repeatability of the prediction model.

### External validation cohort

External validation was also conducted in this study. The external data sets were obtained from the Department of Neurosurgery of Huichang County People's Hospital, a single comprehensive medical center that serves more than 700,000 people in China. From April 2016 to March 2019, 80 patients underwent primary DC due to craniocerebral injury. By querying the follow-up records of these patients and applying the current study protocols, 74 patients were finally included in the external validation cohort.

### Outcome variable

The GOS was used to obtain an assessment of the quality of life. According to the follow-up results, the status of each patient at the end of the observation period was recorded and evaluated according to the GOS(15), of which a score of 1 was death and a score from 2 to 5 represented different survival states from an unfavorable to a favorable outcome. Accordingly, the patients were divided into two groups: the patients with a score of 1–2 were placed in the 6-month unfavorable prognosis group, whereas patients with scores of 3, 4, and 5 were included in the 6-month favorable prognosis group.

### Study variables and definition

(1) The demographic baseline data that characterized the study population included age and sex.

(2) The clinical features included the Glasgow Coma Scale (GCS) score and pupil status at the time of DC, abrupt changes soon after admission, hypoxia before the operation, and injury severity score (ISS) (16) of each patient.

(3) Imaging features obtained from the computerized tomography (CT) scans of each patient included subdural hematoma, epidural hematoma, cerebral contusion, subarachnoid hemorrhage, and the status of the cistern. The status of the cistern was defined as either partially or completely effaced.

(4) Surgical data included operative blood loss, intraoperative hypotension, and postoperative hematoma.

(5) We classified the laboratory auxiliary examination results according to the most common clinical classification and relevant literature, including hemoglobin (HB) (g/L), fibrin (FIB) (g/L), activated partial thromboplastin time (APTT) (s), blood glucose (mmol/L), and blood calcium (mmol/L) (16–19).

### Nomogram construction and validation

The data from the training cohort (*n* = 274) were used to perform univariate and multivariate analyses to identify the independent predictive factors for a poor outcome and formulate a nomogram. Then, the internal (*n* = 117) and external (*n* = 74) validation cohorts were used to validate the nomogram in terms of discrimination and calibration. The discriminative power of the nomogram was evaluated by the area under the receiver operating characteristic (ROC) curve (AUC). The nomogram was calibrated by assessing the concordance between the predicted and observed probabilities. In the present study, calibration plots were constructed to evaluate the goodness-of-fit of the nomogram.

### Data statistical analysis

All continuous variables in the present study had non-normal distributions and thus are expressed as medians (with 25th and 75th quartiles), while categorical variables are presented as frequencies (percentages). For non-normally distributed variables, the Mann–Whitney U-test was used, and for categorical variables, the chi-square test was used when appropriate.

A univariate analysis was conducted for the training cohort to test potential predictive factors, and odds ratios (ORs) and their 95% confidence intervals (CIs) were calculated. Any variables found to be significant (*p* < 0.05) or nearly significant (*p* <0.2) were qualified for the multivariate analysis.

To facilitate the interpretation of the results, we transformed the continuous variables into categorical variables. According to our clinical experience and values in the literature, some variables, such as Hb, FIB, APTT, serum glucose, and serum calcium, were dichotomized using cutoff points of 10 g/dL, <2 g/L, >36 s, >200 mg/dL, and <2.1 mmol/L, respectively, while the GCS score ( ≤ 5, 5–8, >8) was considered a tricategorical variable (18–24). The best cutoff values for ISS and blood loss were 25 and 750 ml, respectively, according to the maximum Youden's index obtained from ROC curve analysis. Due to the inconsistent classification of age in each study, after analyzing the segmented effect of the relationship between age and prognosis and referencing the CRASH model (25), we finally classified age into eight levels: <20, 20–24, 25–29, 30–34, 35–44, 45–54, 55–64, and ≥65. To assess the independent predictors for an unfavorable 6-month neurological outcome, a multivariate logistic regression model (Forward: LR) was further built.

An individualized nomogram model for predicting the 6-month outcome was then constructed based on the results of multivariate logistic regression in the training cohort. To evaluate the discriminability and calibration of the model, data from the internal and external cohorts were applied to the nomogram. The discriminability of the nomogram was assessed in terms of the AUC with a 95% CI (26). The value of the AUC should fall between 0.5 and 1, in which an AUC>0.75 is considered favorable discrimination. To evaluate the calibration of the nomogram, bootstrap-corrected calibration plots were generated. In view of some limitations of the Hosmer–Lemeshow test, in the present study, we directly observed the concordance between the predicted and observed probabilities (27).

The univariate and multivariate analyses were performed using SPSS (version 20.0, IBM SPSS Statistics). ROC curves were generated using MedCalc software (version 15.8, Belgium). The model was constructed and validated using R for Windows (version 4.0.3, USA).

## Results

### Patient characteristics

After applying the inclusion and exclusion criteria, a total of 391 patients with primary DC were enrolled, of whom 274 and 117 were randomly included in the training and internal validation cohorts, respectively. There were 182 (46.5%) 6-month poor prognosis events during the follow-up period for the entire cohort. The median age at presentation was 47 years (range 35–59.5), and the number of male patients in our study was 316 (80.8%), indicating a sex preference. The median GCS score at the time of DC was 6 (range 4–8). Due to having undergone severe trauma, the patients' ISSs were universally high, with a median score of 26 (range 17–33). Table 1 summarizes patient demographics, clinical characteristics, and surgical and laboratory data for the total, training, and validation cohorts. Additionally, all the indicators were comparable between the training and internal validation groups, which indicated that the randomization protocol was reasonable.

**Table 1:** Baseline characteristics of the total, training, and validation cohorts.

	**Total**	**Training cohort**	**Validation cohort**	***p***
	**(*n* = 391)**	**(*n* = 274)**	**(*n* = 117)**	
Gender (male)	316 (80.8%)	221 (80.7%)	95 (81.2%)	0.901
Age (years)	47 (35–59.5)	47 (35–60)	48(35–58)	0.586
GCS at time of DC (IQR)	6 (4–8)	6 (4–8)	5 (4–8)	0.332
Pupillary status at time of DC				0.144
Both reacting pupils	119 (30.4%)	84 (30.7%)	35 (29.9%)	
One reacting pupil	124 (31.7%)	94 (34.3%)	30 (25.6%)	
Bilateral unreacting pupils	148 (37.9%)	96 (35.0 %)	52 (44.4%)	
Pre-operative anemia (yes)	113 (28.9%)	77 (28.1%)	36 (30.8%)	0.594
Subdural hemorrhage	300 (76.7%)	215 (78.5%)	85 (72.6%)	0.213
Epidural hemorrhage	108 (27.6%)	74 (27%)	34 (29.1%)	0.678
Cerebral contusion	353 (90.3%)	247 (90.1%)	106 (90.6%)	0.89
Subarachnoid hemorrhage	348 (89%)	244 (89.1%)	104 (88.9%)	0.963
Basal cisterns (completely effaced)	129 (33%)	86 (31.4%)	43 (36.8%)	0.301
Operative blood loss (ml, IQR)	700 (500–1200)	700 (400–1000)	800 (500–1200)	0.082
Intraoperative hypotension (yes)	74 (18.9%)	52 (19.0%)	22 (18.8%)	0.968
ISS	26 (17–33)	26 (17–32)	26 (18–33)	0.527
HB (g/L)	134 (120–145)	134.5 (121–147)	133 (119–144)	0.524
FIB (g/L)	1.67 (1.3–2.16)	1.67 (1.3–2.15)	1.62 (1.28–2.18)	0.561
APTT (s)	26.6 (23.6–30.5)	26.6 (23.7–30.2)	27 (23.4–31.1)	0.659
Blood glucose (mmol/L)	9.67 (7.67–11.84)	9.71 (7.68–11.81)	9.6 (7.62–11.98)	0.761
Blood calcium (mmol/L)	2.2 (2.06–2.32)	2.2 (2.07–2.32)	2.18 (2.06–2.31)	0.653
Postoperative hematoma (yes)	40 (10.2%)	30 (10.9%)	10 (8.5%)	0.473
6-month unfavorable prognosis	182 (46.5%)	125 (45.6%)	57 (48.7%)	0.574

*APTT, activated partial thromboplastin time; DC, decompressive craniectomy; FIB, fibrinogen; GCS,Glasgow Score Scale; HB, hemoglobin; ISS, Injury Severity Score; IQR, Interquartile range*.

### Univariable analysis

The results of the univariate analysis in the training cohort are summarized in Table 2, which demonstrate that the majority of the variables, except sex, cerebral contusion, and postoperative hematoma were considered potential predictors for an unfavorable 6-month prognosis (*p* <0.2). Before conducting the multivariate analysis, continuous variables were transformed into categorical variables according to our statistical protocols, as described in detail in the “Methods-Data statistical analysis” section.

**Table 2:** Univariate analysis in the training cohort.

	**Favorable prognosis** **(*n* = 149) n,%**	**Unfavorable prognosis** **(*n* = 125) *n*,%**	**OR (95%CI)**	***p***
Age (years)	44 (33–56)	52 (39–64)	1.024 (1.010–1.039)	0.001
Gender (male)	121 (81.2%)	100 (80.0%)	0.926 (0.508-1.688)	0.801
GCS at time of DC (IQR)	8 (6–10)	4 (4–5)	0.505 (0.428–0.597)	<0.001
Pupillary status at time of DC				<0.001
Both reacting pupils	72 (48.3%)	12 (9.6%)	1	
One reacting pupil	60 (40.3%)	34 (27.2%)	3.400 (1.619–7.140)	0.001
Bilateral unreacting pupils	17 (11.4%)	79 (63.2%)	27.882 (12.466–62.366)	<0.001
Pre-operative anemia (yes)	15 (10.1%)	62 (49.6%)	8.792 (4.643–16.648)	<0.001
ISS	21 (17–26)	29 (26–41)	1.143 (1.101–1.187)	<0.001
Subdural hemorrhage	106 (71.1%)	109 (87.2%)	2.764 (1.467–5.205)	0.002
Epidural hemorrhage	50 (33.6%)	24 (19.2%)	0.470 (0.269–0.824)	0.008
Cerebral contusion	137 (91.9%)	110 (88.0%)	0.642 (0.289–1.429)	0.278
Subarachnoid hemorrhage	126 (84.6%)	118 (94.4%)	3.077 (1.273–7.437)	0.013
Basal cisterns (completely effaced)	12 (8.1%)	74 (59.2%)	16.565 (8.313–33.010)	<0.001
Operative blood loss (ml,IQR)	600 (400–800)	800 (500–1500)	1.001 (1.001–1.002)	<0.001
Intraoperative hypotension (yes)	4 (2.7%)	48 (38.4%)	22.597 (7.854–65.015)	<0.001
Postoperative hematoma (yes)	16 (10.7%)	14 (11.2%)	1.048 (0.490–2.242)	0.903
HB (g/L)	135 (125–149)	130 (112–142)	0.985 (0.975–0.996)	0.009
FIB (g/L)	1.78 (1.48–2.16)	1.56 (0.97–2.05)	0.681 (0.500–0.929)	0.015
APTT (s)	25.5 (23.0–28.4)	27.6 (25.3–34.0)	1.084 (1.043–1.126)	<0.001
Blood glucose (mmol/L)	8.99 (7.40–10.77)	10.46 (8.25–12.95)	1.178 (1.086–1.276)	<0.001
Blood calcium (mmol/L)	2.22 (2.10–2.32)	2.19 (1.99–2.32)	0.209 (0.096–0.877)	0.028

*APTT, activated partial thromboplastin time; DC, decompressive craniectomy; FIB, fibrinogen; GCS, Glasgow Score Scale; HB, hemoglobin; ISS, Injury Severity Score; IQR, Interquartile range; OR, odds ratio; CI, confidence interval*.

### Multivariable analysis and nomogram development

**Figure 1:**
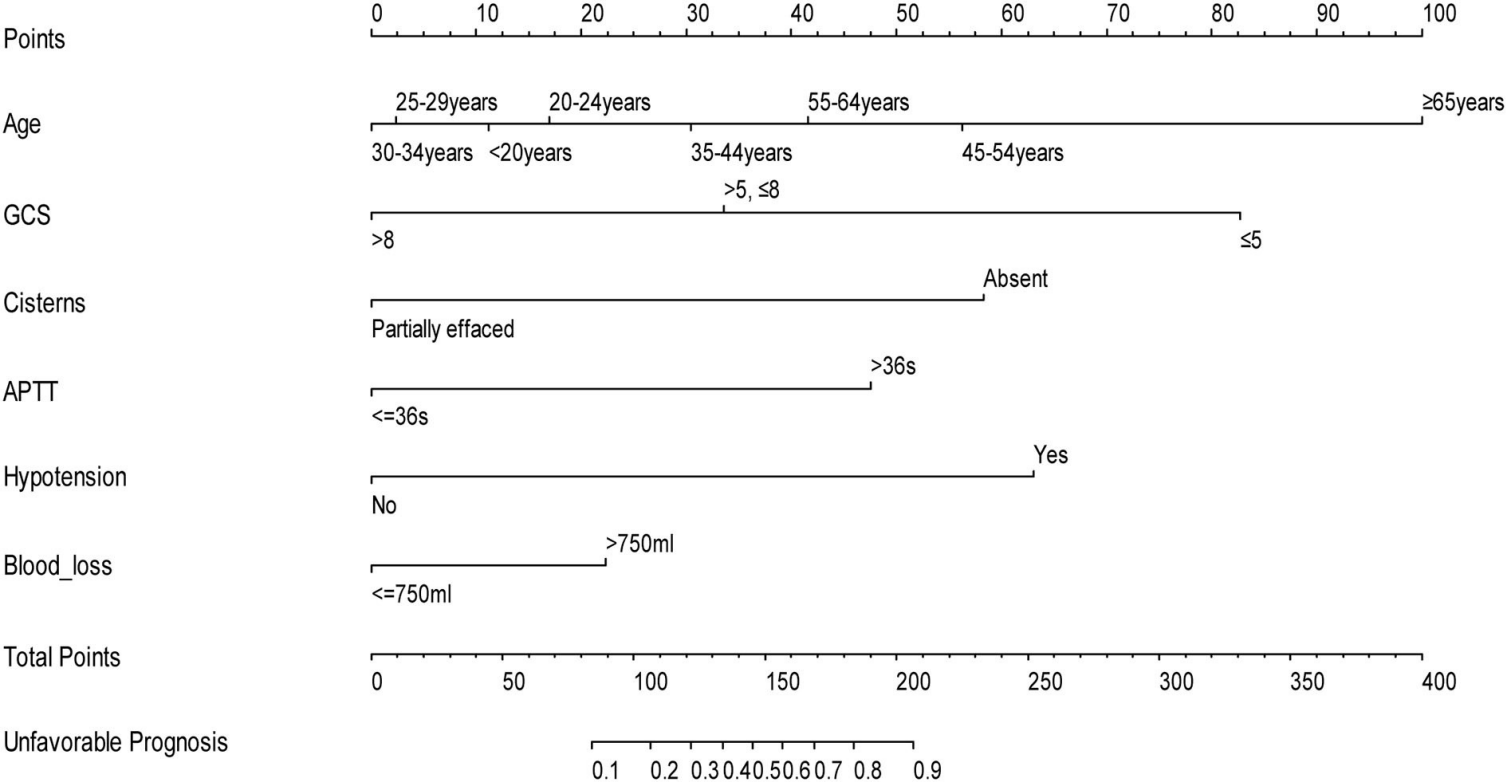
Nomogram to predict the probability of 6-month prognosis in patients with TBI undergoing primary DC (when using this nomogram, the value of each status of the patient will be located on each variable axis, and a vertical line will be drawn to determine the score of each variable value. The total score of the sum of all variable values is on the total score axis. The total score axis corresponds to the probability of a poor prognosis).

Table 3 summarizes the results of the multivariate logistic regression analysis, which identified age (*p* = 0.001), GCS score (*p* < 0.001), operative blood loss of >750 ml (*p* = 0.045), completely effaced basal cisterns (*p* < 0.001), intraoperative hypotension (*p* = 0.001), and APTT>36 (*p* = 0.012) as significant predictors of an unfavorable 6-month prognosis. These independent risk factors were then incorporated to construct a prognostic nomogram for the early assessment of the 6-month prognosis (Figure 1). As shown in the nomogram, a score was obtained for each predictor, which was then summarized as the total score. The predicted risk corresponding to the total score is the probability of an overall 6-month unfavorable prognosis after primary DC.

**Table 3:** Multivariate logistic regression analysis in the training cohort.

**Variables**		**Multivariate results (logistic regression, Forward:LR)**	
		***p*** **-value**	**OR (95% CI)**
Age (years)		0.001	
	20–24	0.843	1.232 (0.156–9.707)
	25–29	0.809	0.728 (0.055–9.583)
	30–34	0.763	0.670 (0.060–9.430)
	35–44	0.458	1.993 (0.323–12.305)
	45–54	0.074	5.038 (0.854–29.767)
	55–64	0.237	2.976 (0.488–18.168)
	≥65	0.001	24.114 (3.707–156.876)
GCS	<0.001	
	>5 ≤ 8	0.071	3.332 (0.902–12.308)
	≤ 5	<0.001	19.391 (5.169–72.738)
Operative blood loss(>750 ml)		0.045	2.223 (1.019–4.853)
Basal cisterns (completely effaced)		<0.001	8.062 (3.299–19.699)
Intraoperative hypotension (yes)		0.001	9.562 (2.527–36.178)
APTT>36 s		0.012	5.488 (1.452–20.740)

*APTT, activated partial thromboplastin time; OR, odds ratio; CI, confidence interval*.

### Internal and external validation of the nomogram

**Figure 2:**
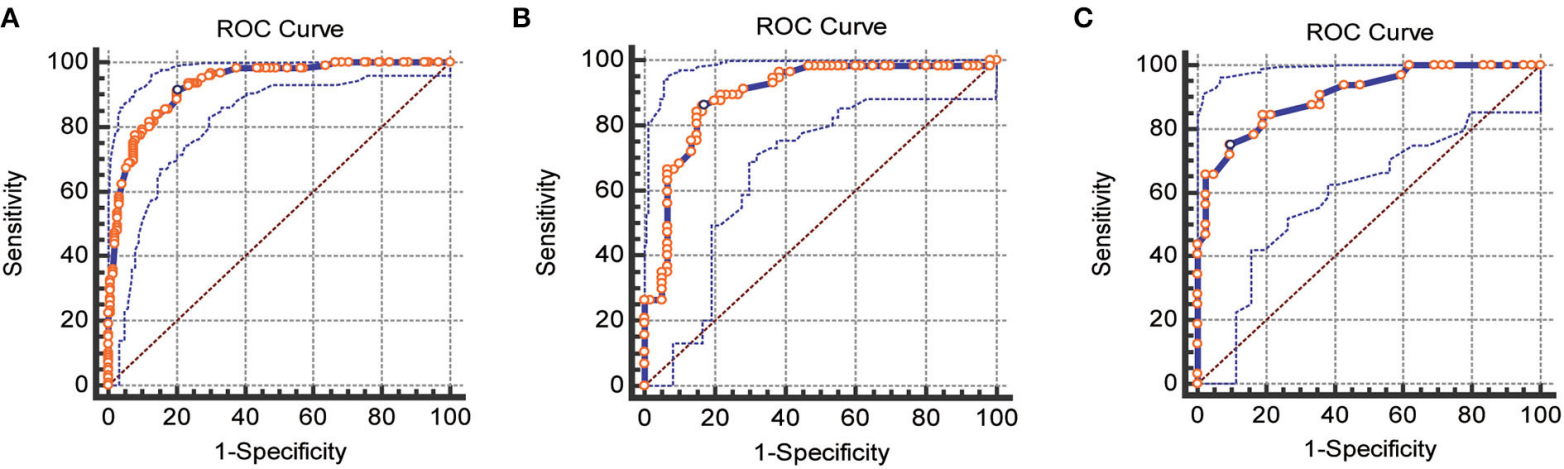
ROC curves for the training, internal, and external validation cohorts, respectively. **(A)** Training cohorts **(B)** internal validation cohorts **(C)** external validation cohorts (AUC = 0.93 vs. 0.89 vs. 0.90).

**Figure 3:**
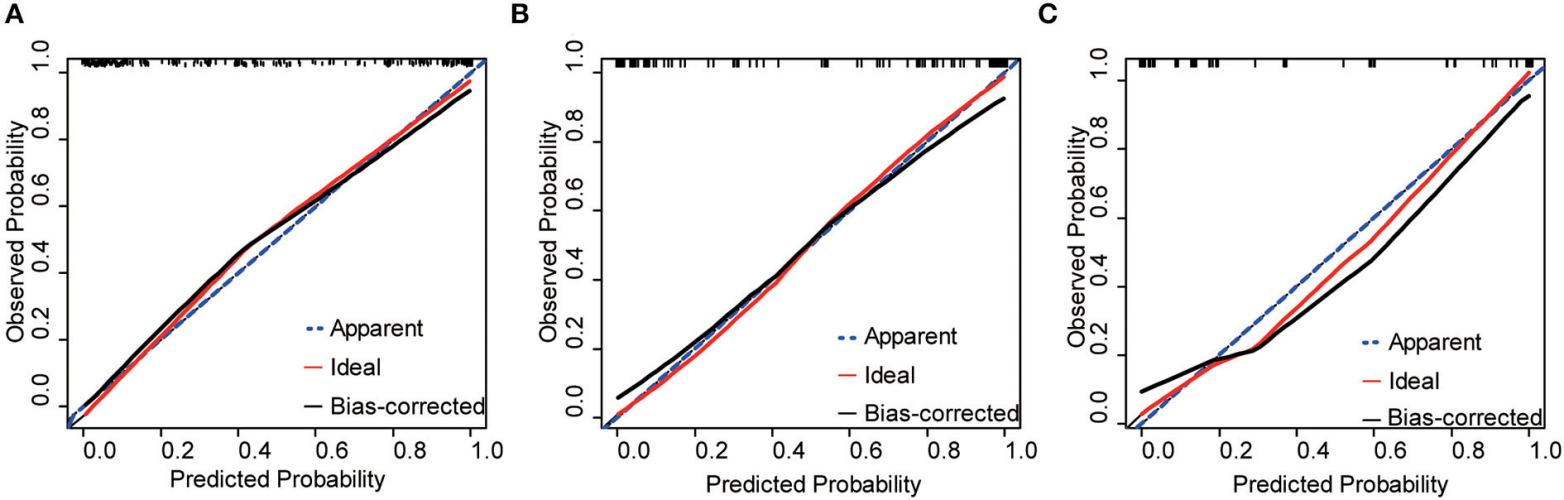
Calibration plots of the nomogram for the training, internal, and external validation cohorts, respectively. **(A)** Training cohorts **(B)** internal validation cohorts **(C)** external validation cohorts.

The nomogram was internally and externally validated based on the discriminability and calibration ability of the model. The discriminability of the nomogram was estimated in terms of the AUC with 95% CI. ROC curves were drawn for the training, internal, and external validation cohorts (Figure 2). The AUCs for the training, internal, and external validation cohorts were 0.93 (95% CI, 0.89–0.96, *p* < 0.0001), 0.89 (95% CI, 0.82–0.94, *p* < 0.0001), and 0.90 (95% CI, 0.84–0.97, *p* < 0.0001), respectively, which indicated that our prediction model had excellent discriminability for the 6-month prognosis after primary DC and an ideal concordance in discrimination for both the training and validation cohorts. The calibration ability of the model is exhibited with bootstrap-corrected calibration plots (Figure 3), which show an optimal concordance between the predicted and actual probabilities of a 6-month unfavorable prognosis in both the training and validation cohorts.

## Discussion

Despite a lack of nationwide epidemiological statistics, a large number of deaths and disabilities caused by TBI have become a serious public health threat in China (28). However, the high mortality of primary DC and the economic burden of the long-term nursing of patients with severe disability or an irreversible vegetative status have led to endless problems for the patient's family and society (8, 29). Due to the lack of well-matched prediction models, currently, we must evaluate the prognostic risk of patients with primary DC and consider how to implement effective regimens to substantially ameliorate the prognosis beyond an initial phase, generally relying on personal experience. At present, only two mainstream prediction models have been published for the prognosis of patients with TBI: the IMPACT model and the CRASH model (25, 30). However, these two models do not specifically focus on TBI patients with DC. Consequently, we aimed to establish an early, accurate, objective, and convenient prediction model for severe TBI patients with primary DC based on clinical indicators after admission.

This study constructed a risk prediction nomogram, a clear tool representing the prediction model, which could be directly applied in the clinic. Before establishing the prediction model, we determined age, GCS score, operative blood loss, completely effaced basal cisterns, intraoperative hypotension, and APTT as significant predictors of an unfavorable 6-month prognosis in patients with TBI after primary DC.

In terms of the multivariate analysis of our study, the correlation between age ≥65 years old and poor prognosis was extremely strong (OR = 24.114), which indicated that elderly patients are more prone to an unfavorable prognosis than young patients. This conclusion is similar to that published in the literature. Paldor et al. (31) performed a retrospective chart review of patients with TBI older than 65 years old submitted to DC and found that <2% had a good outcome 6 months after discharge, while three-fourths of the elderly patients who died were in a vegetative state or were severely disabled. Barthélemy et al. (32) conducted a systematic review of the TBI-related literature between 2011 and 2015 and concluded that younger patients had significantly lower mortality and higher GOS scores than older patients after evaluating the 6-month and 1-year mortality and the GOS score at discharge, taking into account the risk of bias in the RCT. We believe that a substantial portion of elderly patients experience underlying diseases, which tend to deteriorate after DC and evolve into serious postoperative complications, ultimately affecting the prognosis (33). Additionally, neuroplasticity in aged individuals is inferior to that of younger individuals, as is the tolerability of some post-traumatic complications, such as ischemia, hypoxia, and shock. These factors may significantly reduce the probability of recovery from severe trauma in elderly patients even well-after the trauma.

In our study, the GCS score was one of the predictors of an unfavorable 6-month prognosis, especially when it was <5, and had a strong correlation (OR = 19.391) with the catastrophic outcome. A very low GCS score (3 or 4) often indicates that brain stem function is life-threateningly compromised and is generally accompanied by unfavorable imaging features, such as an effaced basal cistern, which was another predictor for an unfavorable 6-month prognosis in our study. Once the signs of constriction or occlusion of the cisterna pontis and/or cisterna ambiens appear on CT, the patient's intracranial pressure will eventually increase. An extremely high ICP will lead to a decrease in intracranial vascular compliance and a sharp reduction in cerebral blood flow. When the cerebral perfusion pressure is lower than 50 mmHg, regardless of the duration, critical consequences could develop (34). Even if these patients survive, it can be difficult for them to recover to a favorable condition. However, some studies have found that a lower GCS score does not necessarily lead to adverse outcomes, especially in pediatric patients. The unimaginable rehabilitation effect may be achieved by prompt decompression and the doctors' confidence (35).

The influence of blood loss on the prognosis of patients undergoing DC has been mentioned occasionally in the literature, indicating that it may primarily affect mortality in pediatric patients. In a study based on a series of children (<15 years old) undergoing DC, the authors found that blood loss was one of the paramount elements in determining prognosis; when the blood loss was >300 ml, the prognosis was significantly worse (36). Desgranges also observed similar results, where among pediatric patients, when the intraoperative blood loss was over 50% of the blood volume, coagulation indexes, such as the international normalized ratio (INR), were significantly higher than with less blood loss (37). In the present study, we observed that intraoperative blood loss was an independent predictor of prognosis in both adults and children. Since DC carries a high risk of bleeding, a large amount of intraoperative blood loss will worsen the hemodynamics during the perioperative period, resulting in a significant increase in the probability of a poor prognosis. Thus, we suggest that the control of intraoperative blood loss is of great importance in the prognosis of patients and thus is deserving of attention from every surgeon.

Some studies have attempted to describe the mechanism by which hypotension after TBI increases in-hospital mortality (38, 39). We observed that intraoperative hypotension was strongly correlated with an unfavorable 6-month prognosis in multivariate analysis (OR: 9.562, *p* = 0.001). We believe that this may be the result of the following: (1) some patients with severe craniocerebral injury were hypotensive before surgery due to blood loss, traumatic shock, and other reasons. If there is too much blood loss during DC, the patient's hypotension will gradually progress to intractable intraoperative hypotension; (2) the rapid release of intracranial pressure when the dura mater is opened, causing a sudden change in sympathetic tension and a sharp decrease in vascular compliance; (3) obstruction of vascular access, which may hinder the resuscitation of blood volume (40); and (4) hemodynamic instability, especially hypotension during the operation, leading to serious insufficient cerebral perfusion. Even if the patient survives, a series of secondary brain injuries can occur (37).

Some previous studies have reported that abnormal coagulation function is highly correlated with an unfavorable prognosis after TBI (23, 41). In the multivariate analysis of our study, among the various coagulation-related factors, only APTT was an independent predictor of an unfavorable 6-month prognosis (OR = 5.488, *p* = 0.012). APTT reflects the comprehensive activity of the endogenous coagulation pathway, which is related to the main coagulation factors, such as VII, XI, VIII, and IX. A prolonged APTT after trauma is caused by activation of the coagulation pathway and the consumption or dysfunction of plasma coagulation factors, which often indicates that the patient is in a hypercoagulable state after the initial period of injury (41). In view of the urgency requiring primary DC, it is necessary to rectify abnormal coagulation function as soon as possible with procedures such as fresh plasma infusion and administration of coagulation factors. This requires not only the attention of doctors but also the cooperation of all departments of the trauma center.

Based on the above factors, we constructed a risk prediction nomogram and validated it with internal and external datasets, demonstrating the practical applicability of the model. In this study, the internal and external validation datasets were used to assess the discriminability and calibration ability, respectively, of the model. Models are often internally validated with homologous data to demonstrate their repeatability, while external validation is used to examine the portability and generalization of the model (27). After importing the internal and external datasets, the AUC of the model remained above 0.85, while the nomogram-predicted probabilities fairly well-matched the observed probabilities, which indicated that the model for predicting the overall 6-month prognosis of patients with TBI after primary DC had a favorable goodness-of-fit, robustness, and predictive power. It is noteworthy that from the calibration plot for internal validation, the calibration curve deviates slightly downward from the reference line when the prediction probability is over 60%, which showed a slight risk overestimation. From the calibration plot of external validation, the performance of external validation is similar to that of internal validation, especially when the prediction probability is higher than 30% and lower than 80%, that is, also a minor overestimation of risk, and this bias should be taken into account when using the model.

However, in clinical practice, the model should be used as an “early warning signal” to remind doctors to pay attention to risk factors, especially controllable factors such as hypotension and abnormal coagulation function, to minimize the probability of an adverse prognosis. We should also note that the prediction model cannot replace the doctor's clinical judgment. For instance, controversy remains regarding whether certain high-risk patients (low GCS score with bilateral dilated pupils, for example) should be actively undergoing DC. We need to use the prediction model to assist the patient's family members in making decisions without violating the principles of treatment and medical ethics.

Several limitations of this investigation must be addressed. This is a single-center retrospective study, thus, there will be unavoidable selection bias. However, we strictly formulated the inclusion and exclusion criteria, which made the data we collected more homogeneous and truly reflective of the actual situation. Additionally, we will conduct a multicenter study in the future, which has been supported by another fund. Another limitation is that we did not include ICP data in the study. Given the large number of patients with TBI and the relative shortage of ICP equipment, our ICP monitor gives priority to patients undergoing secondary DC. ICP monitors were only used for a few patients with primary DC, and thus the associated data may have been significantly biased.

## Conclusion

We constructed a prediction model for the 6-month unfavorable prognosis for patients with TBI undergoing primary DC based on early clinical indicators. The internal and external validation results of the prediction model were encouraging. The prediction model was displayed in the form of a nomogram, which was used to evaluate the prognosis of such patients early, accurately, objectively, and conveniently, and can help to enhance the early identification of high-risk patients.

## Data Availability Statement

The raw data supporting the conclusions of this article will be made available by the authors, without undue reservation.

## Ethics Statement

The studies involving human participants were reviewed and approved by Clinical Research Ethics Committee of Ganzhou People's Hospital. Written informed consent to participate in this study was provided by the participants' legal guardian/next of kin.

## Author contributions

RY designed this study. ZT, KH, MZo, MZh, and QH performed data collection. WW conducted the statistical analysis. ZT and QJ drafted the manuscript. All authors contributed to the article and approved the submitted version.

## Funding

This study was funded by the Science and Technology Department of Jiangxi Province (No. 20203BBGL73174) and the Ganzhou Municipal Health Committee (No. 2022-2-039).

## Conflict of interest

The authors declare that the research was conducted in the absence of any commercial or financial relationships that could be construed as a potential conflict of interest.

## Publisher's note

All claims expressed in this article are solely those of the authors and do not necessarily represent those of their affiliated organizations, or those of the publisher, the editors and the reviewers. Any product that may be evaluated in this article, or claim that may be made by its manufacturer, is not guaranteed or endorsed by the publisher.
